# Wound Healing Effect of Nonthermal Atmospheric Pressure Plasma Jet on a Rat Burn Wound Model: A Preliminary Study

**DOI:** 10.1093/jbcr/irz120

**Published:** 2019-07-11

**Authors:** Yoonje Lee, Sanjaya Ricky, Tae Ho Lim, Ki-Seok Jang, Hongjung Kim, Yeongtak Song, Sang-You Kim, Kyu-sun Chung

**Affiliations:** 1 Department of Emergency Medicine, College of Medicine, Korea University, Seoul, Korea; 2 Department of Translational Medicine, College of Medicine, Hanyang University, Seoul, Korea; 3 Department of Emergency Medicine, College of Medicine, Hanyang University, Seoul, Korea; 4 Convergence Technology Center for Disaster Preparedness, Hanyang University, Seoul, Korea; 5 Department of Pathology, College of Medicine, Hanyang University, Seoul, Korea; 6 Department of Engineering, College of Engineering, Hanyang University, Seoul, Korea

## Abstract

Worldwide, an estimated 6 million patients seek medical attention for burns annually. Various treatment methods and materials have been investigated and developed to enhance burn wound healing. Recently, a new technology, plasma medicine, has emerged to offer new solutions in wound care. As the development of plasma medicine has shown benefit in wound healing, we aimed to assess the effects of plasma medicine on burn wounds. To investigate the effectiveness of a nonthermal atmospheric pressure plasma jet (NAPPJ) for burn wound treatment on a brass comb burn wound rat model. Burn wounds were made by applying a preheated brass comb (100°C) for 2 minutes, which resulted in four full-thickness burn wounds separated by three interspaces. Interspaces were exposed to NAPPJ treatment for 2 minutes and morphological changes and neutrophil infiltration were monitored at 0, 4, and 7 days post-wounding. The percentage of necrotic interspace was higher in the control group than in the plasma-treated group (51.8 ± 20.5% vs 31.5 ± 19.0%, *P* < .001). Moreover, the exposure of interspace to NAPPJ greatly reduced the number of infiltrating neutrophils. In addition, the percentage of interspace that underwent full-thickness necrosis in the plasma-treated group was smaller than that in the control group (28% vs 67%). NAPPJ exposure on interspaces has a positive effect on burn wounds leading to wound healing by reducing burn injury progression.

Globally, there are approximately 6 million patients seeking burns medical service per year.^1^ Burn injury is a common type of traumatic injury, causing considerable morbidity and mortality. Furthermore, burn wound care is one of the most expensive treatment modalities, due to the cost needed for long hospital stay and rehabilitation, as well as wound and scar treatment.^2,3^ In order to hasten burned wound healing and epithelialization and to prevent infection and wound progression to the deeper skin layers, adequate burn wound care is necessary.^4^ For this purpose, various treatment methods and materials have been studied and developed. However, evidence-based methods and materials for burn wound care are still not widely available. Therefore, burn wound care is one of the most challenging research sectors of medicine in this 21st century.^5^

Recently, a new technology, plasma medicine, has emerged to offer new solutions in wound care. “Plasma” refers to the fourth state of matter. When a gas is heated or subjected to high electrical force, electrons are accelerated, resulting in collision with the atoms and molecules, knocking off and freeing other electrons, and thus creating positively charged atomic and molecular ions. This mixture of uncharged atoms, molecules, and ions, as well as electrons, is what is referred to as “plasma.” The collision of these electrons with the air substance such as O_2_, H_2_O, N_2_, etc. will eventually generate reactive species and are utilized for medical pursposes.^5–8^ Few applications of plasma have been explored, including bacterial inactivation, blood coagulation, wound healing, tissue regeneration, cancer treatment, dentistry, and chronic ulcer treatment.^5,8^ Moreover, plasma was proved to effectively reduce populations of many types of bacteria and yeasts.^9,10^

Plasma also contributes to wound healing by inducing cell proliferation,^11^ migration of keratinocytes and fibroblasts for wound re-epithelialization,^12–14^ as well as by inducing wound healing-related genes.^15^ Plasma effectiveness for healing of common cutaneous and chronic ulcer wounds has been previously reported.^8^ Based on these reported plasma benefits in wound healing, we were intrigued about the effect of plasma medicine on third-degree burn wounds, as the process of burn injury differs from that of other injuries. Furthermore, to the best of our knowledge, no previous study has assessed the effects of plasma treatment on third-degree burn wounds.

In this study, we investigated the effectiveness of a nonthermal atmospheric pressure plasma jet (NAPPJ) in third-degree burn wounds treatment on a brass comb burn wound rat model.

## METHODS

### Study Setting and Animal Subjects

This prospective randomized longitudinal rat study was approved by the institutional animal care and use committee at Hanyang University (approval number HY-IACUC-18-0010). The study was carried out in the Division of Laboratory Animal Research of our institution. Six male Sprague-Dawley rats (weight, 400–450 g) were used in this study. The rats had access to standard food and water ad libitum, and were acclimatized to the environment for several days prior to the experiment. Cages were managed in accordance with the National Research Council guidelines.^16^

### Characteristics of Plasma Device

**Figure 1.**
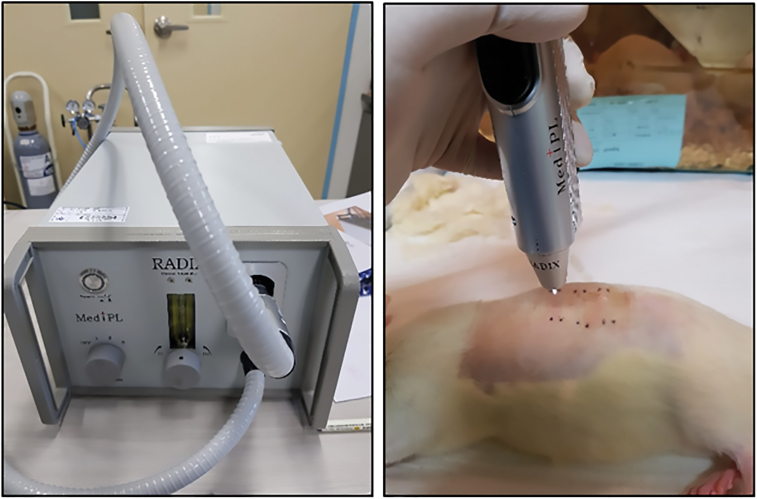
Nonthermal atmospheric pressure plasma jet (left) and its application on unburned interspace (right).

The NAPPJ used in this experiment was developed by MediPL in Korea. The RADIX plasma generator (MediPL, Seongnam city, Republic of Korea) used in this study is a microwave power signal generator (Figure 1). This device ejects a single plasma jet from a pen-shaped resonator with an operating frequency of 50/60 Hz. The working gas for the plasma generator was argon, delivered at a rate of 5.0 standard liters per minute (slm) and the input power was 2.5 Watt. The plasma produced by this generator formed a stable bright plume that was ~7 mm in length. The temperature produced by this plasma generator was less than 40°C, and emitted less than 0.05 ppm ozone, thus being eco-friendly.

### Burn Model

This study used brass combs with four prongs separated by three 5 mm notches that would produce three unburned sites (interspaces) between the four burned sites. The interspaces were not directly injured, but within hours they would progress to ischemia and undergo necrosis (at approximately 24–48 hours). The interspaces represented the zones of stasis, whereas the burned sites represented the zones of coagulation. The dimensions of the brass comb were 20 × 25 × 55 mm with four 10 × 25 mm rectangular prongs, separated by three 5-mm-wide grooves.

### Experimental Protocols

This experiment was based on Adam Singer’s previously established rat contact thermal injury model.^17^ The rats were anesthetized by inhalation of 0.5% to 2.5% isoflurane. The hair on their back was clipped using an electric clipper. The brass comb (200 g) was preheated in a water bath at 100°C for 3 minutes and then perpendicularly applied to the back skin without pressure for a period of 90 seconds. This resulted in four full-thickness burns separated by three unburned interspaces. Rats were randomly grouped into two groups: a control group and a plasma-treated group. Argon gas flow was used (5 slm) and the input power of the plasma generator was 2.5 Watt. The interspaces of the plasma-treated group were treated with NAPPJ for 2 minutes at a 2 cm distance between the tip of NAPPJ and the rat skin (Figure 1). Elizabethan collars were used to prevent the rats from licking or scratching the wounds. The treatments were reapplied on day 1, 2, 3, 4, 5, 6, and 7. All animals were then euthanized by CO_2_ inhalation 7 days after injury.

### Measures and Outcomes

Wounds were observed and photographed daily for necrotic evidence of the interspaces showed by the formation of scabs. At day 7 after injury, the interspace areas were excised and analyzed for histomorphology.

Histopathological studies were performed by fixing tissue samples on formalin overnight at 4°C; subsequently they were dehydrated in a graded series of ethanol (70%, 80%, 90%, and 100% ^v^/_v_). Then, the samples were embedded in paraffin and sectioned at 4 µm thickness, deparaffinized, and stained with hematoxylin and eosin.

The outcomes were collected at 1 hour, 4 days, and 7 days after the injury and they were assessed blindly. Based on the histological result, damaged areas reaching below dermis area were considered as third-degree burn wound (necrotic tissue). Based on gross visualization, the percentage of unburned interspace area that progress to full-thickness necrosis was measured and analyzed using Image Color Summarizer (Martin Krzywinski, Genome Science Centre, Vancouver, BC, Canada). Lastly, the number and infiltration of inflammatory cells until certain area was observed.

### Statistical Analysis

Statistical analysis was conducted using Minitab 17 software (Minitab Inc., State College, PA). The mean burn wound progression percentage between plasma and control group were compared using Student’s *t*-test. Sample size equation is done with the formula: *E* = *n*_sample_ − *n*_group_.^18^ Data were expressed as mean ± *SD* and *P*-values < .05 were considered statistically significant. *n*_sample_, total number of interspace samples (*n*_sample_ = 36); *n*_group_, total number of groups (*n*_group_ = 2).

## RESULTS

A total of 36 interspaces were created in six rats and randomly assigned to two groups of three rats.

### Gross Findings

**Figure 2.**
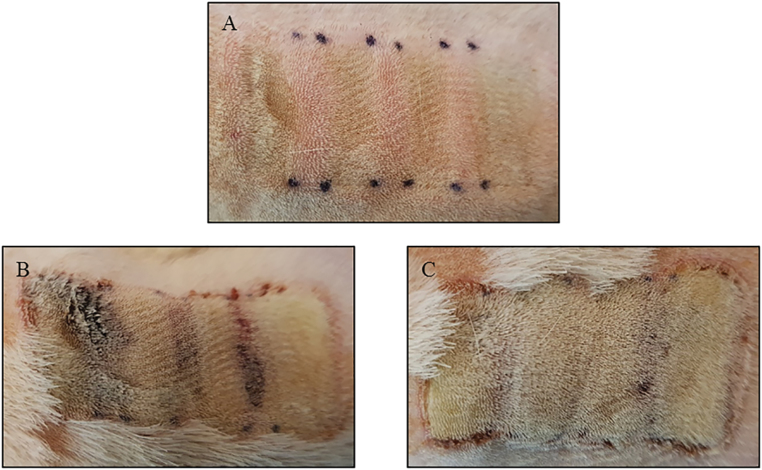
Gross appearance of comb burn wound on nontreated skin and treated skin. Appearance of burn wound immediately after its creation (A) and 7 days after its creation without plasma treatment (B) and with plasma treatment (C). Four rectangular burn wounds are separated by three unburned interspaces that are marked with black dots.

NAPPJ suppressed necrosis progression in interspaces caused by burn wounds (Figure 2). Based on gross visualization, the percentage of interspace area that progressed to full-thickness necrosis in the control group was higher than that of the group exposed to NAPPJ (51.8 ± 20.5% vs 31.5 ± 19.0%, *P* < .001) (Table 1). NAPPJ treatment of interspace area had a significant effect on wound healing.

**Table 1.** Full-thickness necrotic interspace number and percentage of burn wound progression

Total Interspace	Control (*n* = 18)	Plasma-Treated (*n* = 18)	*P*
Full-thickness necrotic interspace	12 (67%)	5 (28%)	
The percentage of the unburned interspace that became necrotic	51.8 ± 20.5	31.5 ± 19.0	<.001

Categorical data are expressed as number (percentage) and are compared by Student’s *t*-test.

### Histological Findings

**Figure 3.**
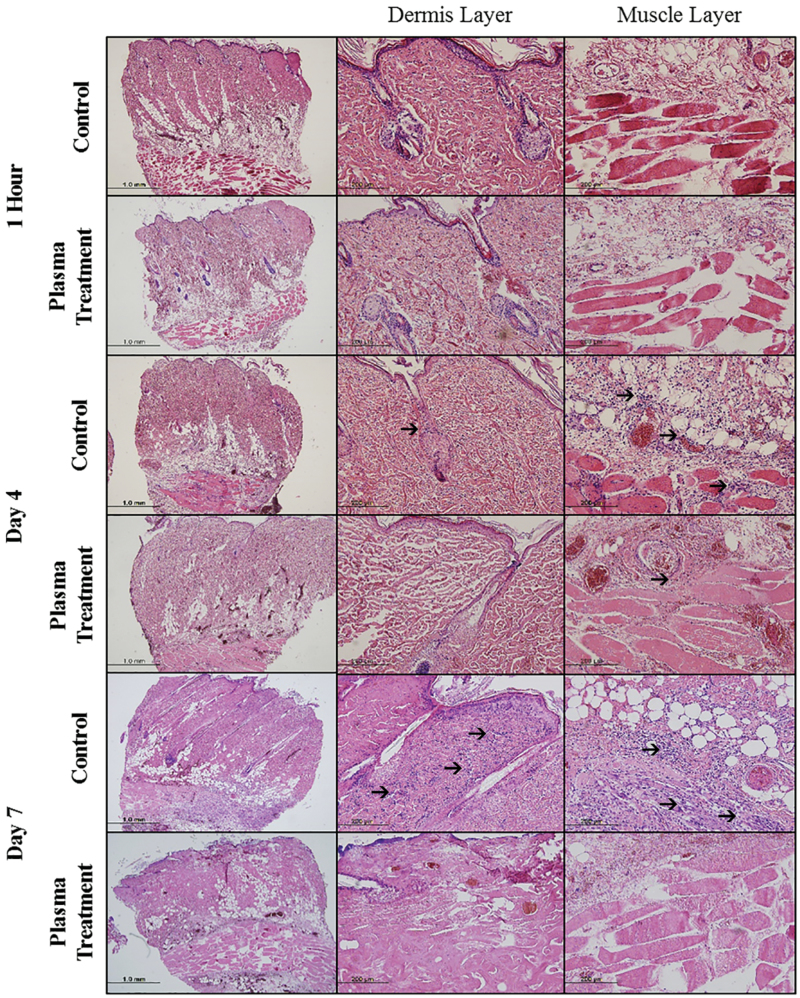
Histological appearance of necrotic interspaces in the control group and in the plasma-treated group 1 hour, 4 days, and 7 days after injury. Arrows indicating infiltrating inflammatory cells (purple). One rat each for 1 hour and 4 days assessment and 3 rats for 7 days assessment were used.

On histology, burn progression in the interspaces was assessed. Interspace biopsies of both groups were taken 1 hour post-injury and no evidence of injury was observed. Similar results were obtained for interspace biopsies that were taken from both control and plasma-treated groups at 4 days post-injury. At 4 days after injury, a few neutrophils were observed beneath the muscle layer in the interspaces of both groups. No significant differences were found between the control and the plasma-treated groups. However, the neutrophil infiltration in muscle and dermal layers was higher in the interspaces of the control group. The observation continued until 7 days post-injury and significant differences were observed between the two groups. At 7 days after injury, a large number of infiltrating neutrophils dominating the muscle and dermal layers were noted in the interspaces of the control group. In contrast, very few neutrophils were detected in the muscle layer of the plasma-treated interspace group (Figure 3).

### Morphological Findings

Thickening of the dermal layer was observed in both groups. Dermal thickness was related to the increase in the number of fibroblasts, as well as the deposition of collagen fibers in the dermis in response to injury. The number of interspaces that progressed to full-thickness necrosis, indicated by dermis thickening, was 67% (12 out of 18) in the control group. In comparison, animals that received NAPPJ treatment showed a lower rate of full-thickness conversion (28%, 5 out of 18) (Table 1). These results suggested that treating interspaces surrounded by the burn wound with NAPPJ had positive effects on burn wound healing.

## DISCUSSION

The main findings of the present study were that the NAPPJ treatment by using argon as the working gas led to the prevention of the interspace underwent to necrotic tissue by reducing the inflammatory phase in a rat comb burn wound model. As a result, the interspaces of rats treated with NAPPJ treatment were grossly and microscopically less progressed to the third-degree burn wound compared to the interspaces of the rats from the control group.

Wound healing is a complex process in which the skin and the underlying tissues repair themselves after injury.^19^ The process of wound healing is depicted in a discrete timeline of physical phases constituting the posttrauma repairing process. In undamaged skin, the epidermis and dermis form a protective barrier against the external environment. When this barrier is broken, a regulated sequence of biochemical cascades is set into motion to repair the damage.^19,20^ This process is divided into four predictable phases: blood clotting (hemostasis), inflammation, tissue growth (proliferation), and tissue remodeling (maturation). Generally, blood clotting may be considered to be part of the inflammation phase, instead of a separate phase.^21^

Burn wounds heal through the same process as general wounds. However, burn wounds have different characteristics from those of general wounds. Burn wounds are characterized by a central zone of cellular necrosis surrounded by a zone of stasis that has a chance to progress to necrosis.^17,22^ Necrosis is defined by swelling and rupturing of the cells, leading to water and extracellular ion influx that impairs homeostasis. In addition, interruption of homeostasis can result in excessive production of cytokines that can attenuate burn wound healing.^23^ Therefore, unlike general wounds, the solution to the problem that is exacerbated by ischemia is the most important part of the treatment or healing of burn wounds.

Recently, the emergence of plasma medicine has attracted the attention of researchers by its versatile applications in the medical field, such as bacterial inactivation, blood coagulation, tissue regeneration, and wound healing. Mild levels of reactive oxygen species (ROS) produced by nonthermal plasma, including hydroxy peroxide (H_2_O_2_) and nitric oxide (NO), have a good effect on the progression of wound healing.^5,8^ When the reactive species were exposed to the skin, it will act as a second messenger that will rely the information that arrives at membrane cell via ligand-receptor binding throughout the cells. This will eventually be received by the cells thus altering their gene expression. Due to the aforementioned reasons, we applied plasma to burn wounds, which has been shown to be effective in promoting cell proliferation and re-epithelialization by its application to acute or chronic general wounds.

The results of the present study showed that plasma treatment reduced the percentage of unburnt ischemic interspaces that developed necrosis after 7 days of injury. Additionally, histopathological findings of plasma-treated interspaces showed more fibroblasts and collagen, and lesser neutrophil infiltration in the muscle layer compared to the untreated interspaces. Reactive species generated by nonthermal plasma might play an important role in reducing the inflammation and stimulating proliferation. Moreover, the effect of plasma treatment on wound healing is in agreement with the results of other studies.^5–8^

Laroussi et al and Sen et al reported that ROS are involved in fibroblast-associated collagen production and in the synthesis of growth factors.^5,24^ In addition, Graves et al showed that ROS are essential for the initial stage of homeostasis through their role in mediating tissue factor (TF-mRNA), platelet recruitment, and platelet activation. Furthermore, H_2_O_2_ is known to act as a second messenger for platelet-derived growth factor, vascular endothelial growth factors, and tissue growth factors (TGF).^6^ A study by Lee et al reported that ROS could increase messenger ribonucleic acid (mRNA) expression of anti-inflammatory cytokines and decrease mRNA expression of pro-inflammatory cytokines.^23^ Moreover, Fathollah et al similarly reported that plasma induces the expression of TGF-β.^11^ Thus, the aforementioned results emphasized the positive effects of plasma on burn wound healing.

It has been stated that not only ROS, but also NO is produced by nonthermal plasma. NO plays a major role in wound healing by creating a class of molecules termed electrophilic biomolecules. One of the most important type of electrophilic biomolecules formed by reactive nitrogen species are nitrated fatty acids (NO_2_-FAs). Groeger et al showed that NO_2_-FAs appeared to have valuable therapeutic benefit by inducing anti-inflammatory gene expression, improving vascular function, and attenuating pro-inflammatory polymorphic neutrophils and macrophage function.^25^ It is to be noted that in the past, reactive species were thought to be the hallmarks of oxidative stress; however, they are currently considered to be potent anti-inflammatory molecules. Of course, excessive concentration of reactive species, including NO, can lead to cell-cycle arrest, senescence, and apoptosis. Nevertheless, some researchers found that lower or mild levels of NO tend to favor growth and act to oppose apoptosis.^26,27^ This explains the high number of neutrophils infiltrating the control group’s interspaces up to the dermis layer and their absence in the plasma-treated group’s interspaces.

Generally, silver sulfadiazine has been known as the gold standard for burn wound treatment.^28^ However, it has some disadvantages, such as delayed and incomplete re-epithelialization, limited penetration to the depth of the wound, and ineffectiveness against some bacteria.^29^ Some studies on other topical treatment agents have also been conducted. For instance, a research conducted by El-Kased et al assessed the effect of honey on burn wound treatment. They showed that honey could induce burn wound healing; however, the property of honey to liquefy at skin burn temperature restricts its direct application. Because of this reason, authors incorporated honey in a hydrogel formula for better application on burn wounds.^30^ The use of curcumin on burn wounds was also reported by El-Refaie et al. However, curcumin has a similar delivery problem to that of honey. Therefore, a hyaluronic acid-based gel-core hyaluosomes loaded with curcumin was developed for better delivery of curcumin.^31^ Our plasma-based treatment application on burn wound used reactive species that can penetrate the tissue easily and promote the expression of genes responsible for wound healing. The application of plasma on burn wound can become a potent therapy in burn wound care.

This study had several limitations that should be mentioned. First, as this study was conducted in rats, its results may not be generalized to humans because of anatomical and physiological differences. However, some effects of plasma for treatment of burn wounds could be confirmed in this study. Therefore, this method can be applied as an adjuvant treatment modality in burn wound care. Second, the validated comb burn model used in this study is a primarily a tool to study horizontal injury progression; therefore, it may not resemble the vertical progression of burn in typical human burn injury. Third, the sample size seems to be small in this study. However, in previous study using brass comb burn wound model, the author had designed that each interspace is counted as a single outcome. Therefore, in this study the sample size was 18 samples each group. Additionally, the quantification of necrotic cells was performed by visual quantification of the percentage of burned interspace at 7 days post-injury. No molecular marker of cell death was used to quantify the number of necrotic cells. Therefore, based on these preliminary data, a future study should use molecular markers for wound healing to further investigate the effect of plasma in burn wound care.

## CONCLUSIONS

This study assessed the effects of nonthermal atmospheric pressure plasma jet on the brass comb burn wound model in rats. This study found that plasma reduced the burn injury progression in the unburned interspaces in a rat comb burn model.

## Notes

Authors’ Contributions: S.R. and Y.L. contributed equally to this study. Y.L. mainly designed this study. S.R. and Y.L. conducted this study. T.H.L. supervised the process of this study and data analysis. K.-S.J. blindly conducted the histopathological examination and analysis. S.R. and Y.L. wrote the initial draft of the article. H.K. and Y.S. contributed to critical revision of the manuscript for important intellectual content. S.-Y.K. and K.-S.J. contributed by setting and using of plasma device for wound care. All authors extensively reviewed and approved the final version of the article. This manuscript has not been submitted or published elsewhere in whole or in part.

Funding: His research was supported by the National Research Foundation of Korea (NRF) funded by the Ministry of Science, ICT & Future Plannig (NRF-2018M3A9F7062526). The fund did not participate in the study design, collection, analysis, interpretation, writing of the report, and decision to submit article for publication.

## References

[1] Brusselaers N, Monstrey S, Vogelaers D, Hoste E, Blot S Severe burn injury in Europe: a systematic review of the incidence, etiology, morbidity, and mortality. Crit Care 2010;14:R188. 20958968 10.1186/cc9300 PMC3219295

[2] Sánchez JL, Perepérez SB, Bastida JL, Martínez MM Cost-utility analysis applied to the treatment of burn patients in a specialized center. Arch Surg 2007;142:50–57. 17224500 10.1001/archsurg.142.1.50

[3] de Roche R, Lüscher NJ, Debrunner HU, Fischer R Epidemiological data and costs of burn injuries in workers in Switzerland: an argument for immediate treatment in burn centres. Burns 1994;20:58–60. 8148079 10.1016/0305-4179(94)90108-2

[4] Campos LS, Mansilla MF, de la Chica AMM Topical chemotherapy for the treatment of burns. Rev Enferm 2005;28:67–70. 15981974

[5] Laroussi M, Kong M, Morfill G, Stolz W. Plasma medicine: application of low-temperature gas plasmas in medicine and biology. New York, NY: Cambridge University Press; 2012.

[6] Graves DB The emerging role of reactive oxygen and nitrogen species in redox biology and some implications for plasma applications to medicine and biology. J Phys D: Appl Phys 2012;45:263001.

[7] Sagai M, Bocci V Mechanisms of action involved in ozone therapy: is healing induced via a mild oxidative stress? Med Gas Res 2011;1:29. 22185664 10.1186/2045-9912-1-29 PMC3298518

[8] Fridman G, Friedman G, Gustol A, Shekhter AB, Vasilets VN, Fridman A Applied plasma medicine, plasma. Process Polym 2008;5:503–533.

[9] Kamgang-Youbi G, Herry JM, Meylheuc T, et al. Microbial inactivation using plasma-activated water obtained by gliding electric discharges. Lett Appl Microbiol 2009;48:13–18. 19170858 10.1111/j.1472-765X.2008.02476.x

[10] Traylor MJ, Pavlovich MJ, Karim S, et al. Long-term antibacterial efficacy of air plasma-activated water. J Phys D: Appl Phys 2011;44:472001.

[11] Fathollah S, Mirpour S, Mansouri P, et al. Investigation on the effects of the atmospheric pressure plasma on wound healing in diabetic rats. Sci Rep 2016;6:19144. 26902681 10.1038/srep19144 PMC4763329

[12] Schmidt A, Bekeschus S, Wende K, Vollmar B, von Woedtke T A cold plasma jet accelerates wound healing in a murine model of full-thickness skin wounds. Exp Dermatol 2017;26:156–162. 27492871 10.1111/exd.13156

[13] Kang SU, Kim YS, Kim YE, et al. Opposite effects of non-thermal plasma on cell migration and collagen production in keloid and normal fibroblasts. PLoS One 2017;12:e0187978. 29145520 10.1371/journal.pone.0187978 PMC5690474

[14] Xu GM, Shi XM, Cai JF, et al. Dual effects of atmospheric pressure plasma jet on skin wound healing of mice. Wound Repair Regen 2015;23:878–884. 26342154 10.1111/wrr.12364

[15] Barton A, Wende K, Bundscherer L, et al. Non-thermal plasma increases expression of wound healing related genes in a keratinocyte cell line. Plasma Med 2013;3:125–136.

[16] National Academy of Sciences. Guide for the care and use of laboratory animals. Washington, DC: National Academy Press; 1996.

[17] Singer AJ, Taira BR, Lin F, et al. Curcumin reduces injury progression in a rat comb burn model. J Burn Care Res 2011;32:135–142. 21088615 10.1097/BCR.0b013e318203337b

[18] Charan J, Kantharia ND How to calculate sample size in animal studies? J Pharmacol Pharmacother 2013;4:303–306. 24250214 10.4103/0976-500X.119726 PMC3826013

[19] Orgill D, Blanco C. Biomaterials for treating skin loss. Cambridge, UK: Woodhead Publishing Limited; 2009.

[20] Rieger S, Zhao H, Martin P, Abe K, Lisse TS The role of nuclear hormone receptors in cutaneous wound repair. Cell Biochem Funct 2004;33:1–13. 10.1002/cbf.3086 PMC4357276 25529612

[21] Stadelmann WK, Digenis AG, Tobin GR Physiology and healing dynamics of chronic cutaneous wounds. Am J Surg. 1998;176(2A Suppl):26S–38S. 9777970 10.1016/s0002-9610(98)00183-4

[22] Contassot E, Gaide O, French LE Death receptors and apoptosis. Dermatol Clin 2007;25:487–501. 17903608 10.1016/j.det.2007.06.010

[23] Lee OJ, Ju HW, Khang G, et al. An experimental burn wound-healing study of non-thermal atmospheric pressure microplasma jet arrays. J Tissue Eng Regen Med 2016;10:348–357. 26227832 10.1002/term.2074

[24] Sen CK, Khanna S, Babior BM, Hunt TK, Ellison EC, Roy S Oxidant-induced vascular endothelial growth factor expression in human keratinocytes and cutaneous wound healing. J Biol Chem 2002;277:33284–33290. 12068011 10.1074/jbc.M203391200

[25] Groeger AL, Freeman BA Signaling actions of electrophiles: anti-inflammatory therapeutic candidates. Mol Interv 2010;10:39–50. 20124562 10.1124/mi.10.1.7 PMC3139380

[26] Thomas DD, Ridnour LA, Isenberg JS, et al. The chemical biology of nitric oxide: implications in cellular signaling. Free Radic Biol Med 2008;45:18–31. 18439435 10.1016/j.freeradbiomed.2008.03.020 PMC2572721

[27] Dröge W Free radicals in the physiological control of cell function. Physiol Rev 2002;82:47–95. 11773609 10.1152/physrev.00018.2001

[28] Hosseini SV, Tanideh N, Kohanteb J, Ghodrati Z, Mehrabani D, Yarmohammadi H Comparison between alpha and silver sulfadiazine ointments in treatment of Pseudomonas infections in 3rd degree burns. Int J Surg 2007;5:23–26. 17386910 10.1016/j.ijsu.2006.03.007

[29] Hussain S, Ferguson C Best evidence topic report: silver sulphadiazine cream in burns. Emerg Med J 2003;23:929–932. 10.1136/emj.2006.043059 PMC2564257 17130603

[30] El-Kased RF, Amer RI, Attia D, Elmazar MM Honey-based hydrogel: in vitro and comparative in vivo evaluation for burn wound healing. Sci Rep 2017;7:9692. 28851905 10.1038/s41598-017-08771-8 PMC5575255

[31] El-Refaie WM, Elnaggar YS, El-Massik MA, Abdallah OY Novel curcumin-loaded gel-core hyaluosomes with promising burn-wound healing potential: development, in-vitro appraisal and in-vivo studies. Int J Pharm 2015;486:88–98. 25818063 10.1016/j.ijpharm.2015.03.052

